# Immuno-surveillance and protection of the human cochlea

**DOI:** 10.3389/fneur.2024.1355785

**Published:** 2024-05-16

**Authors:** Wei Liu, Hao Li, Charlotta Kämpfe Nordström, Niklas Danckwardt-Lillieström, Sumit Agrawal, Hanif M. Ladak, Helge Rask-Andersen

**Affiliations:** ^1^Department of Surgical Sciences, Otorhinolaryngology and Head and Neck Surgery, Uppsala University, Uppsala, Sweden; ^2^Department of Otolaryngology-Head and Neck Surgery, Western University, London, ON, Canada; ^3^Department of Medical Biophysics, Western University, London, ON, Canada; ^4^Department of Electrical and Computer Engineering, Western University, London, ON, Canada

**Keywords:** human cochlea, IBA1, immuno-surveillance, super resolution microscopy, synchrotron phase-contrast imaging

## Abstract

**Background:**

Despite its location near infection-prone areas, the human inner ear demonstrates remarkable resilience. This suggests that there are inherent instruments deterring the invasion and spread of pathogens into the inner ear. Here, we combined high-resolution light microscopy, super-resolution immunohistochemistry (SR-SIM) and synchrotron phase contrast imaging (SR-PCI) to identify the protection and barrier systems in the various parts of the human inner ear, focusing on the lateral wall, spiral ganglion, and endolymphatic sac.

**Materials and methods:**

Light microscopy was conducted on mid-modiolar, semi-thin sections, after direct glutaraldehyde/osmium tetroxide fixation. The tonotopic locations were estimated using SR-PCI and 3D reconstruction in cadaveric specimens. The sections were analyzed for leucocyte and macrophage activity, and the results were correlated with immunohistochemistry using confocal microscopy and SR-SIM.

**Results:**

Light microscopy revealed unprecedented preservation of cell anatomy and several macrophage-like cells that were localized in the cochlea. Immunohistochemistry demonstrated IBA1 cells frequently co-expressing MHC II in the spiral ganglion, nerve fibers, lateral wall, spiral limbus, and tympanic covering layer at all cochlear turns as well as in the endolymphatic sac. RNAscope assays revealed extensive expression of fractalkine gene transcripts in type I spiral ganglion cells. CD4 and CD8 cells occasionally surrounded blood vessels in the modiolus and lateral wall. TMEM119 and P2Y12 were not expressed, indicating that the cells labeled with IBA1 were not microglia. The round window niche, compact basilar membrane, and secondary spiral lamina may form protective shields in the cochlear base.

**Discussion:**

The results suggest that the human cochlea is surveilled by dwelling and circulating immune cells. Resident and blood-borne macrophages may initiate protective immune responses via chemokine signaling in the lateral wall, spiral lamina, and spiral ganglion at different frequency locations. Synchrotron imaging revealed intriguing protective barriers in the base of the cochlea. The role of the endolymphatic sac in human inner ear innate and adaptive immunity is discussed.

## Introduction

The human cochlea contains a complex cell machinery capable of converting mechanical vibrations into electric signals, which are then sent to the brain via the auditory nerve. The sensory organ of Corti (OC) consists of sensitive hair cells and a multitude of various specialized supporting cells. Moreover, there are cells that generate an electric field potential in the lateral wall on which receptor function depends. Considering its proximity to infection-prone areas, the human cochlea is highly challenged by acute and chronic middle-ear infections, but it also demonstrates astonishing robustness.

Recent studies have shown that the human inner ear is populated by immune system cells (1–5). This refutes the idea that the human and mammalian inner ear is “immune-privileged” or lacking the ability to mount immune responses (6, 7). Inner ear macrophages appear to be resident but also recruited from blood-borne monocytes from bone marrow myeloid precursors, rather than through self-renewal like brain microglia (8–10). The cells may protect the inner ear from various pathogens, act as scavenger cells, and optimize tissue repair, but they may also cause foreign body responses and potentially cause adverse immune reactions and disease (11–13). Recently, the human cochlear nerve and lateral wall macrophages were revealed to show signs of structural alterations that were highlighted as possible sites of aberrant macrophage activity that could lead to age-related cochlear pathology and hearing loss (14).

Migratory macrophages could shelter the inner ear via surveillance mechanisms through the expression of fractalkine, also known as chemokine (C-X3-C motif) ligand 1, a protein in humans encoded by the CX3CL1 gene. Fractalkine may interact with the macrophage chemokine receptor CX3CR1 to enhance activity (15, 16). Cell-bound chemokine also promotes connection of leukocytes to triggered endothelial cells (17). It has been proposed that spiral ganglion neurons (SGNs) and macrophages could form a neuro-immune link between hair cells and neurons which protects the cochlear nerve under various conditions. Fractalkine chemokine signaling may promote macrophage incursion and survival of auditory neurons after induced hair cell damage. Recently, human studies showed the manifestation of the production of fractalkine gene transcripts in SGNs, spiral lamina regions, and basilar membrane (4). Macrophage function may depend on the location and differences in macrophage morphology and distribution between the apical and basal regions of the cochlea have been described (18). It could explain the different vulnerability of the sensory nerves and receptors in the cochlea (19, 20).

**Figure 1:**
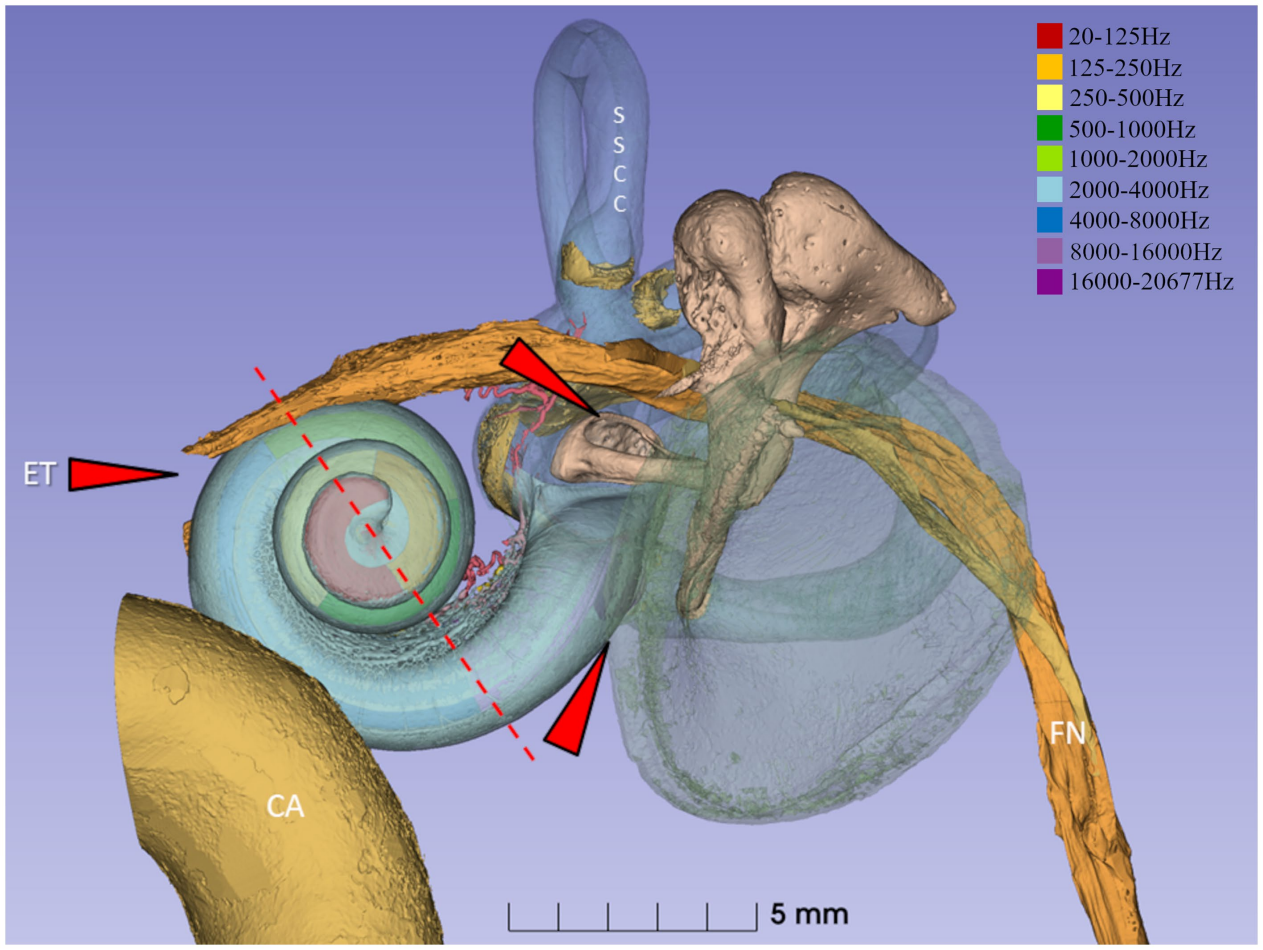
Synchrotron 3D reconstruction of a left human ear. The basilar membrane and spiral ganglion were segmented, and the frequency coordinates were calculated using Greenwood’s formula (21) and dendrite tracing. The red interrupted line denotes the level of sectioning and estimated frequencies in light micrographs 2–6. External pathogens may reach the middle ear and challenge the inner ear function via the Eustachian tube (ET) and the oval and round windows (red arrows). The spiral ganglion and lateral wall are highlighted in the present investigation. SSCC, superior semi-circular canal. CA, carotid artery. FN, facial nerve.

In this study, we extended and correlated prior immunohistochemistry data using both confocal and super-resolution structured illumination microscopy (SR-SIM), with the micro-cellular anatomy of the human cochlea at different frequency locations assessed by synchrotron radiation phase-contrast imaging (SR-PCI, Figure 1). The study aimed to explore more specifically if there are local extrinsic and intrinsic protective features essential for the preservation and maintenance of the human sense of hearing.

### Ethical approval

The study conformed with the Declaration of Helsinki and was approved by the Ethics Review Board (No. 99398, 22/91999, cont., 2003, no. C254/4; no. C45/72007, Dnr. 2013/190) at the Uppsala University Hospital (no. 99308). Written information was given to the patients, and informed consent was obtained. Cadaveric samples used for SR-PCI were obtained with permission from the body bequeathal program at Western University (London, ON, Canada) in accordance with the Anatomy Act of Ontario and Western’s Committee for Cadaveric Use in Research (Approval # 06092020).

## Materials and methods

### Surgically obtained tissue

Cochlear tissue was obtained from patients ranging from 40 to 70 years of age with life-threating petro-clival meningioma and compression of the brainstem where surgical removal of one cochlea was necessary. Data were collected during the time period of 1997–2021 with ethical approval and patient consent. The surgery was performed as a two-stage operation with facial nerve re-routing postero-inferiorly and total petrosectomy, followed by complete tumor removal. The operation time was approximately 15–20 h, and performed by an oto-neuro-surgical team at Uppsala University Hospital. Drilling of the cochlea added an additional 10 min to the total surgery time. The cochleae were dissected out, and a small rim of bony tissue around the cochlea was saved (22, 23). The specimens were immediately placed in fixative in the operating room after surgical removal. There was no evidence that the benign tumor had infiltrated the cochlea. Trans-cochlear surgery was developed as an anterior extension of the trans-labyrinthine approach in the early 1970s without retraction of the brain, after posterior re-routing of the facial nerve and removal of the cochlea and petrous apex (24). The technique is also used for clivus lesions, chordomas, petrous apex cholesteatomas, and epidermoids located anterior to the internal acoustic meatus. The unique preservation offers opportunities to analyze the normal fine structure and gene localization using SR-SIM of the entire cochlear specimen (25). Analyses of the human endolymphatic sac (ES) were based on specimens obtained at surgical labyrinthectomy and removal of vestibular schwannoma during the period of 1990–2020 at Uppsala University Hospital. Specimens were obtained with ethical approval and patient consent. No data on age, gender, or audiometric results were retrieved.

### Light microscopy

The high-resolution light microscopy data presented here (mid-modiolar section) were from one male individual of 50 years of age with normal hearing in both ears (specimen NDL75). The cochlea was fixed in 13.3% fluorocarbon containing fixative in 2% glutaraldehyde solution and 0.05 M sodium phosphate buffer (mixing ratio of 2:1) followed by fixation with 1% osmium tetroxide at 4°C for 4 h (26). This unique specimen was recently used for analyzing the organ of Corti cell architecture at different frequency locations (27, submitted for publication). Thereafter, it was placed in 0.1 mol/L sodium ethylene-diamine tetra-acetic acid (Na-EDTA) for 4 weeks at room temperature. Decalcification was checked by radiography. The tissue was dehydrated and embedded in Epon (Resolution Performance Products, Houston, TX, United States). Semi-thin sections were cut with a glass knife perpendicular to the long axis of the modiolus and stained with toluidine blue. Frequency positions were estimated, and a tonotopic map of the basilar membrane/organ of Corti was created by a nonlinear least squares fitting of a Greenwood-like function to the data of an equally designed cochlea (21, 28, 29) that underwent SR-PCI at the Canadian Light Source Inc. (Saskatoon, SK, Canada) using the Biomedical Imaging and Therapy beamline (05ID-2). The SR-PCI technique used has been previously described (30–32). Open source medical imaging software, 3D Slicer (www.slicer.org, version 5.0.3) (33), was used to segment and create 3D representations of 10 human specimens for analyses of the human round window region, the ES, and its periaqueductal bone marrow. Vascular connections were analyzed potentially supplying the ES with immune competent cells. Moreover, the round window niche was studied for the presence of false or pseudo-membranes that conceivably protect the round window niche and inner ear.

### Immunohistochemistry (SR-SIM)

Immunohistochemistry was performed using super-resolution structured illumination microscopy (SR-SIM) on tissue from seven patients operated for petroclival meningioma. Hearing thresholds (pure tone average (PTA) audiometry) were normal in five patients. In those with hearing loss, one patient 40 years of age had 40–80 dB hearing loss at 1–8 kHz, and one patient 70 years of age had 50 dB hearing loss at 2–4 kHz. The immunohistochemistry procedures used were described in previous publications (1, 2). Briefly, the tissue was fixed in a 4% paraformaldehyde phosphate buffer solution (PBS). The cochleae were decalcified in 10% Na-EDTA solution at pH 7.2 for 4 weeks. They were embedded in Tissue-Tek O.C.T. embedding compound (Polysciences, Inc., Warrington, PA, United States), frozen, and sectioned at 8–10 μm using a cryostat microtome. The sections were incubated with an antibody solution under a humidified atmosphere at 4°C for 20 h. They were then incubated with secondary antibodies conjugated to Alexa Fluor (Thermo Fisher Scientific, Uppsala, Sweden), counterstained with the nuclear stain 4′,6-diamidino-2-phenylindole dihydro-chloride (DAPI), mounted with ProLong Gold Antifade Mountant (Thermo Fisher Scientific), and then covered with cover glass compatible with confocal and super-resolution microscopes. Primary and secondary antibody controls were made to exclude endogenous reaction products (34). Analyses of the human ES were based on over 20 sample specimens obtained at surgical labyrinthectomy using the trans-labyrinthine approach to remove vestibular schwannoma during the period of 1990–2020 at Uppsala University Hospital.

For immunohistochemistry of the ES, no data on age, gender, or audiometric results were retrieved. The surrounding bone was dissected using diamond drills, and a thin shell of bone was saved around the sac. Both the intra- and extra-osseous parts of the ES were analyzed. The endolymphatic duct and the most distal sac on the sigmoid sinus could not be investigated. The tissue was immediately placed in 4% paraformaldehyde in PBS and then placed in 0.5 M Na-ethylene-diamine-tetra-acetic acid (EDTA) solution for decalcification. The ESs were embedded in Tissue-Tek O.C.T. (Polysciences, Inc.) for the frozen sections. The endolymphatic sacs were sectioned at 8–10 μm using a Leica cryostat microtome (35).

### Antibodies

Information about the primary and secondary antibodies is shown in Table 1. The antibody against type IV collagen labeled the basal lamina surrounding Schwann and satellite glia cells, blood vessels, and epithelium. For resident macrophages, we used the antibody against IBA1. Specificity was proven by IBA1 antibody blotting (36). The fractalkine antibody was a monoclonal antibody. This antibody’s specificity was verified in a western blotting experiment (37).

**Table 1:** Antibodies used for studies of the human cochlea and endolymphatic sac.

Primary antibody	Type	Dilution	Host	Catalog number	Producer
IBA1	Polyclonal	1:100	Rabbit	PA5-27436	Thermo Fisher, Waltham, United States
MHC II	Monoclonal	1:100	Mouse	MA5-11966	Thermo Fisher, Waltham, United States
Collagen IV	Polyclonal	1:10	Goat	AB769	Millipore, Burlington, United States
CX3CL1	Monoclonal	1:50	Mouse	MAB3651-100	R&DSystems, Minneapolis, United States
CD11b	Monoclonal	1:50	Rabbit	AB52478	Abcam, Cambridge, United Kingdom
CD4	Polyclonal	1:150	Goat	AF-379-NA	R&DSystems, Minneapolis, United States
CD8α	Monoclonal	1:100	Mouse	MAB1509	R&DSystems, Minneapolis, United States
CD68	Monoclonal	1:50	Mouse	NB100-683	Novus, Littleton, United States
TLR 4	Oligoclonal	1:10	Rabbit	710,185	Thermo Fisher, Waltham, United States
c-Kit/CD117	Polyclonal	1:100	Rabbit	PA5-16770	Thermo Fisher, Waltham, United States
Laminin β2	Monoclonal	1:100	Rat	#05–206	Millipore
Tuj 1	Polyclonal	1:200	Rabbit	#04–1,049	Millipore
P2Y12	Polyclonal	1:50	Rabbit	#PA5-34079	Invitrogen
CCR2	Polyclonal	1:100	Rabbit	NBP1-48337	Novus Biologicals
CCL2	Polyclonal	1:500	Rabbit	NBP1-07035	Novus Biologicals

### RNAscope® protocol

The human protein fractalkine is also known as chemokine (C-X3-C motif) ligand 1, and is encoded by the CX3CL1 gene. Our technique used for detection of CX3CL1 gene transcripts in the human cochlea was recently described (2). The expression and distribution of the ATP1B1 gene transcripts encoding the Na/K-ATPase β1 isoforms in different domains of the cochlea using RNA *in situ* hybridization were also previously outlined (25). These gene transcripts were useful as a control since they are selectively expressed in the marginal cells of the stria vascularis (Table 2).

**Table 2:** Gene information for the RNAscope probe designing or purchase.

Gene name	Species	Gene ID	Chromosome	Location	Producer
CX3CL1	Human	6,376	16q21	411,261	BioTechne

## Results

Histology showed unprecedented preservation of the cell anatomy of the human cochlea including the spiral ganglion, lateral wall, and organ of Corti.

### The spiral ganglion

**Figure 2:**
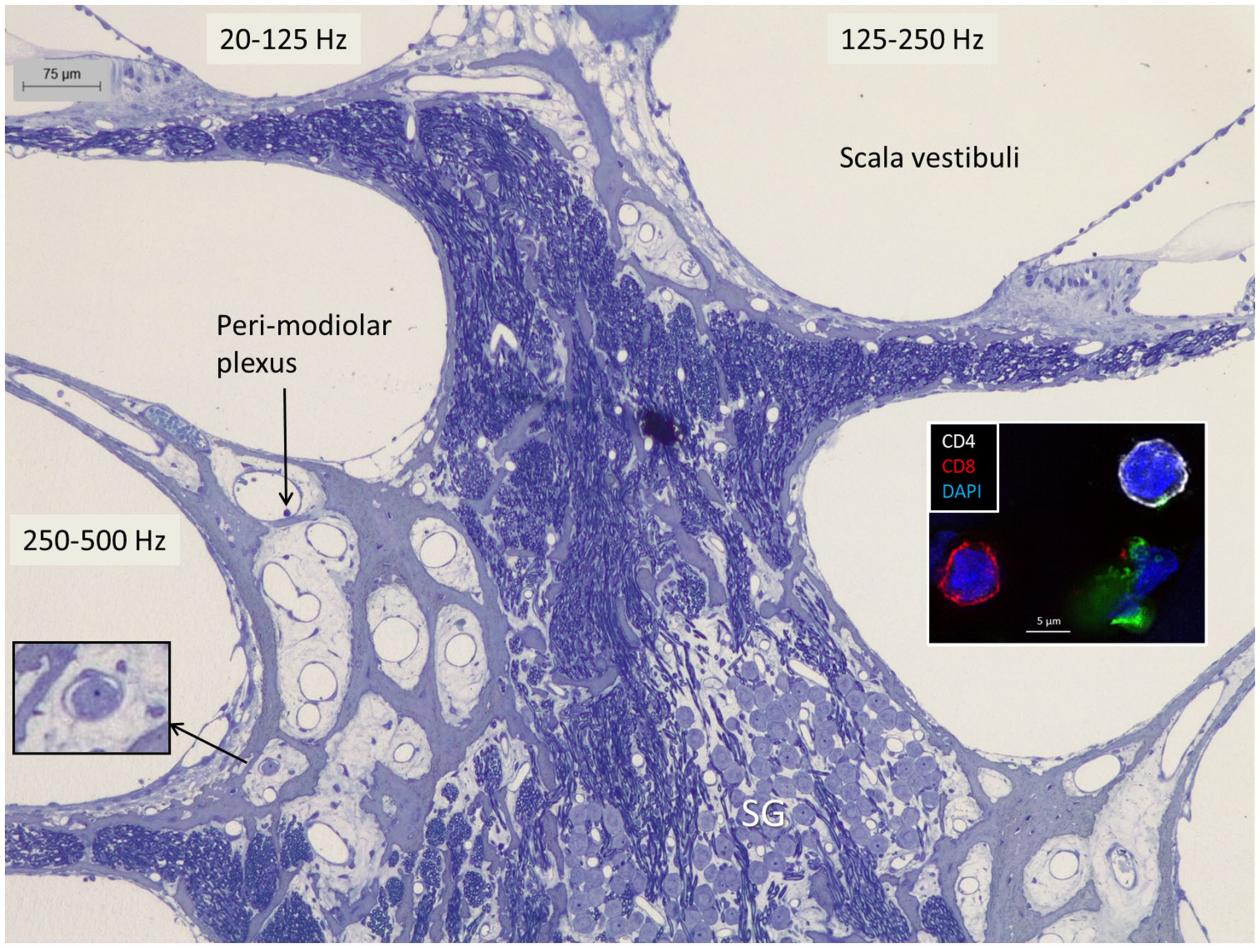
The apical region of the human cochlea is endowed with a perimodiolar thin-walled vascular plexus. Occasionally, small vessels contain white blood cells (left inset). Both the spiral ganglion and nerve fascicles contain macrophages expressing IBA1 as shown in Figure 9A. Right inset shows CD4 and CD8 lymphocytes in the spiral ganglion (3).

**Figure 3:**
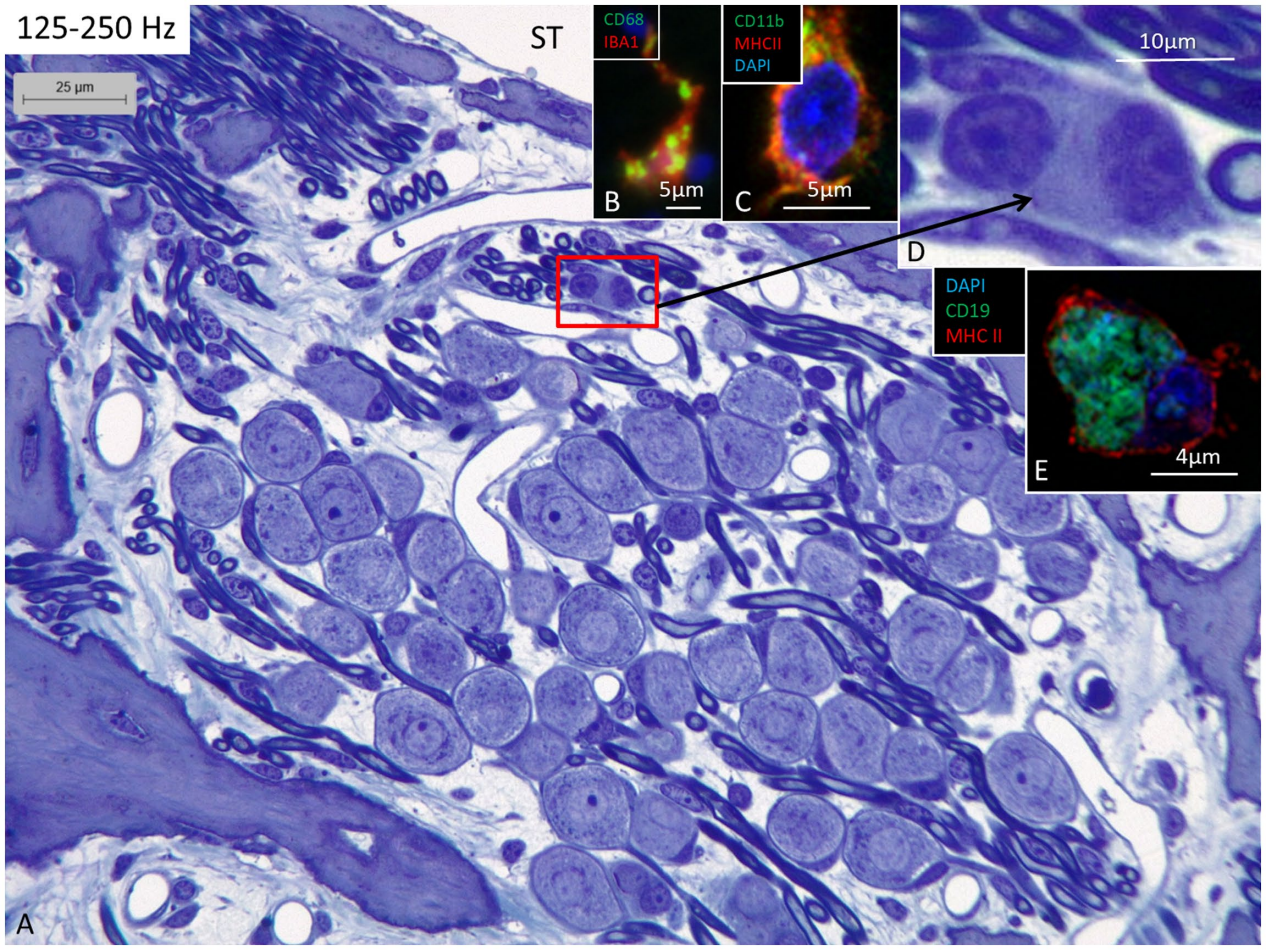
**(A)** Light microscopy of human spiral ganglion neuron cells supplying hair cells in the 125–250 Hz region. The cell bodies are surrounded by many capillaries derived from the perimodiolar plexus and by free mesenchymal cells belonging to the white blood cell lineages, many of which express IBA1. **(B)** Cell in the upper spiral ganglion co-expressing IBA1 and CD68. **(C)** Cell in the upper spiral ganglion expressing MHC II and CD11b. **(D)** Perivascular free cells. **(E)** CD19-positive cells expressing MHC II could occasionally be seen. ST, scala tympani.

**Figure 4:**
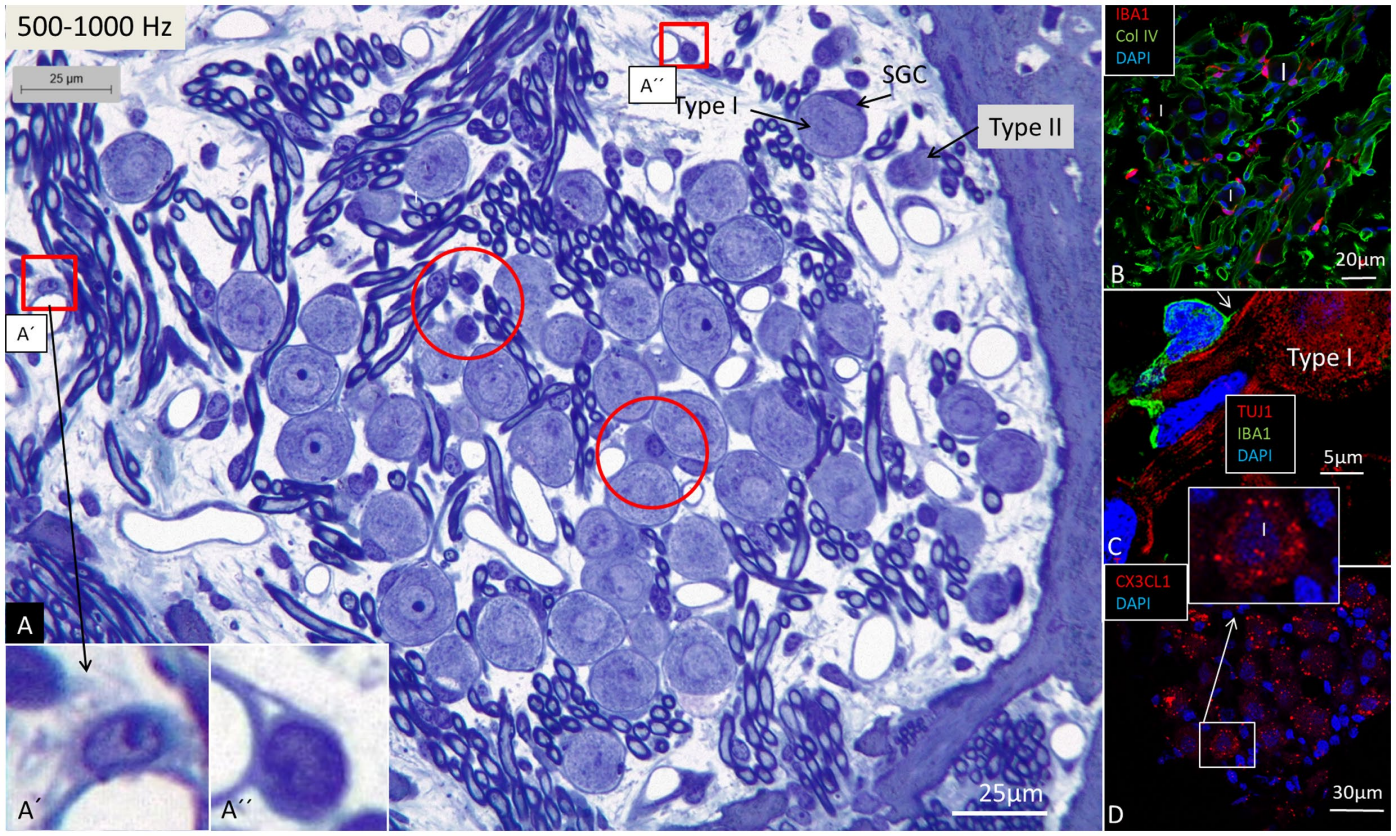
Light microscopy of spiral ganglion neurons (type I and type II) supplying hair cells in the 500–1,000 Hz region. Perivascular dendritic and round cells (framed) are seen as well as free cells located around the type I SGNs (encircled). Framed areas are magnified in **(A’,A”)**. **(B)** Immunohistochemistry shows IBA1 cells closely associated with the type I SGN cell bodies (confocal microscopy). **(C)** An IBA1 cell adheres to the external surface of a type I SGN cell body (SR-SIM). **(D)** Type I SGNs express fractalkine gene transcripts (SR-SIM). I, Type I SGN. SGC, spiral ganglion cell.

**Figure 5:**
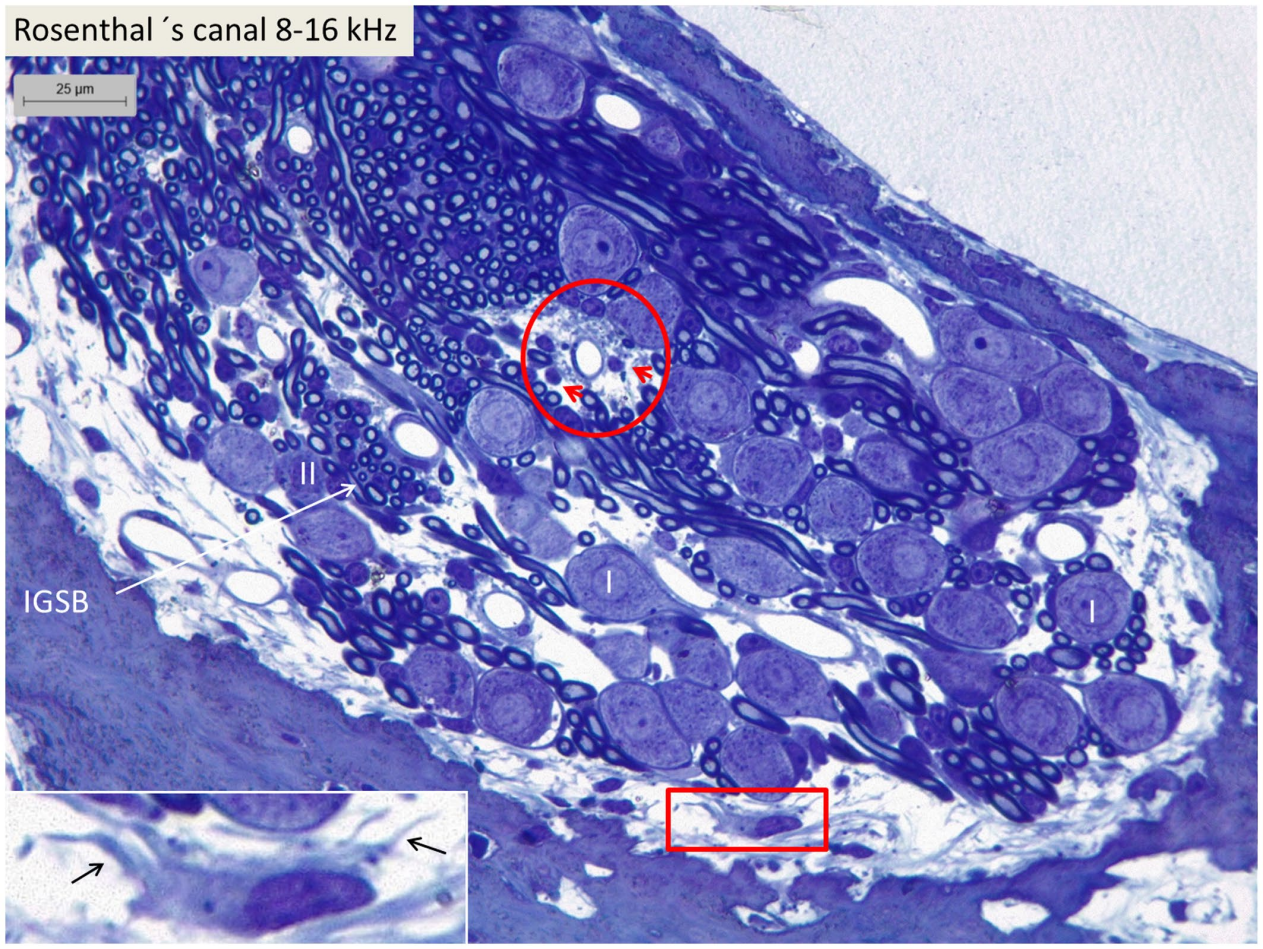
Light microscopy of spiral ganglion neurons in the basal turn (type I and type II) supplying hair cells in the 8–16 kHz region. Perivascular round cells (encircled) and a peripheral dendritic-like cell (framed) can be seen. Inset shows framed area in higher magnification. I, Type I SGN. II, Type II SGN. IGSB, intra-ganglionic spiral bundle.

The SGNs showed minimal fixation artifacts. The upper central modiolus is shown in Figure 2. A perimodiolar plexus with thin-walled blood vessels sent many small tributaries and capillaries centrally, occasionally containing and surrounded by white blood cells. These vessels lacked a recognizable muscular wall. The spiral ganglion reached the scala tympani between the second and third turns at the frequencies of 125–250 Hz, where bundles of myelinated dendrites projected into the spiral lamina. The Rosenthal’s canal (first turn) and central modiolus contained spirally arranged ganglion cell bodies and nerve fibers supplying the three turns (Figures 3–5). The type I SGN cell bodies lacked a surrounding myelin layer, and their large round nuclei contained prominent nucleoli. Type II SGNs and an intra-ganglionic spiral bundle housing efferent nerve fibers could be identified. Apically, nerve cell bodies frequently clustered with cells physically interacting even without an intervening satellite glia cell. Such cell interaction could be noted occasionally also at higher frequencies.

Confocal microscopy and SR-SIM revealed many IBA1-positive macrophages in the lateral wall in all three turns as well as in the entire spiral ganglion. The cells did not express microglia markers TMEM119 and P2Y12. SGNs diffusely expressed fractalkine. A multitude of IBA1 cells interacted physically with both the unmyelinated type I cell bodies and the axonal initial segments as well as the myelinated nerve fibers (Figure 4C). These conditions could be observed at all levels such as the Rosenthal’s canal supplying the first turn and in the central modiolus supplying the second and third turns. Figure 3 shows SGN cell bodies supplying the 125–250 Hz region surrounded by many capillaries branched from perimodiolar vessels. In the upper part of the spiral ganglion, IBA1 cells also expressed CD68 and CD11b (Figures 3B,C). Some cells had a typical sand-glass appearance. Closely related peri-capillary round cells were noted microscopically (Figure 3D), and with immunohistochemistry, CD19-positive B-cells also expressing MHC II were detected occasionally (Figure 3E). SGNs that supplied hair cells in the 500–1,000 Hz region are shown in Figure 4A. Both type I and type II ganglion cell bodies were surrounded by many capillaries. Dendritic-like and monocyte-like cells were located around the blood vessels (Figures 4A’,A’’). Immunohistochemistry showed IBA1 cells closely associated with the type I nerve cell bodies at many locations (Figures 4B,C). SR-SIM of type I spiral ganglion cell bodies expressed fractalkine gene transcripts (Figure 4D). The gene transcripts were mainly expressed in type I ganglion cell bodies as red stained puncta. A few puncta were seen in satellite and Schwann cells in the myelinated axons. SGNs supplying hair cells in the 8–16 kHz region are shown in Figure 5. Rosenthal’s canal was crowded with cell bodies and axon fascicles. Type I and type II spiral ganglion cell bodies were seen as well as the intra-ganglionic spiral bundle containing thin myelinated and unmyelinated efferent nerve fibers. Centrally located capillaries were surrounded by round mono-nuclear cells and peripherally dendritic-like cells (Figure 5, inset). At this level, CD4- and CD8-positive lymphocytes were occasionally identified, mostly in the peripheral region of Rosenthal’s canal as earlier described (3).

### The lateral wall

**Figure 6:**
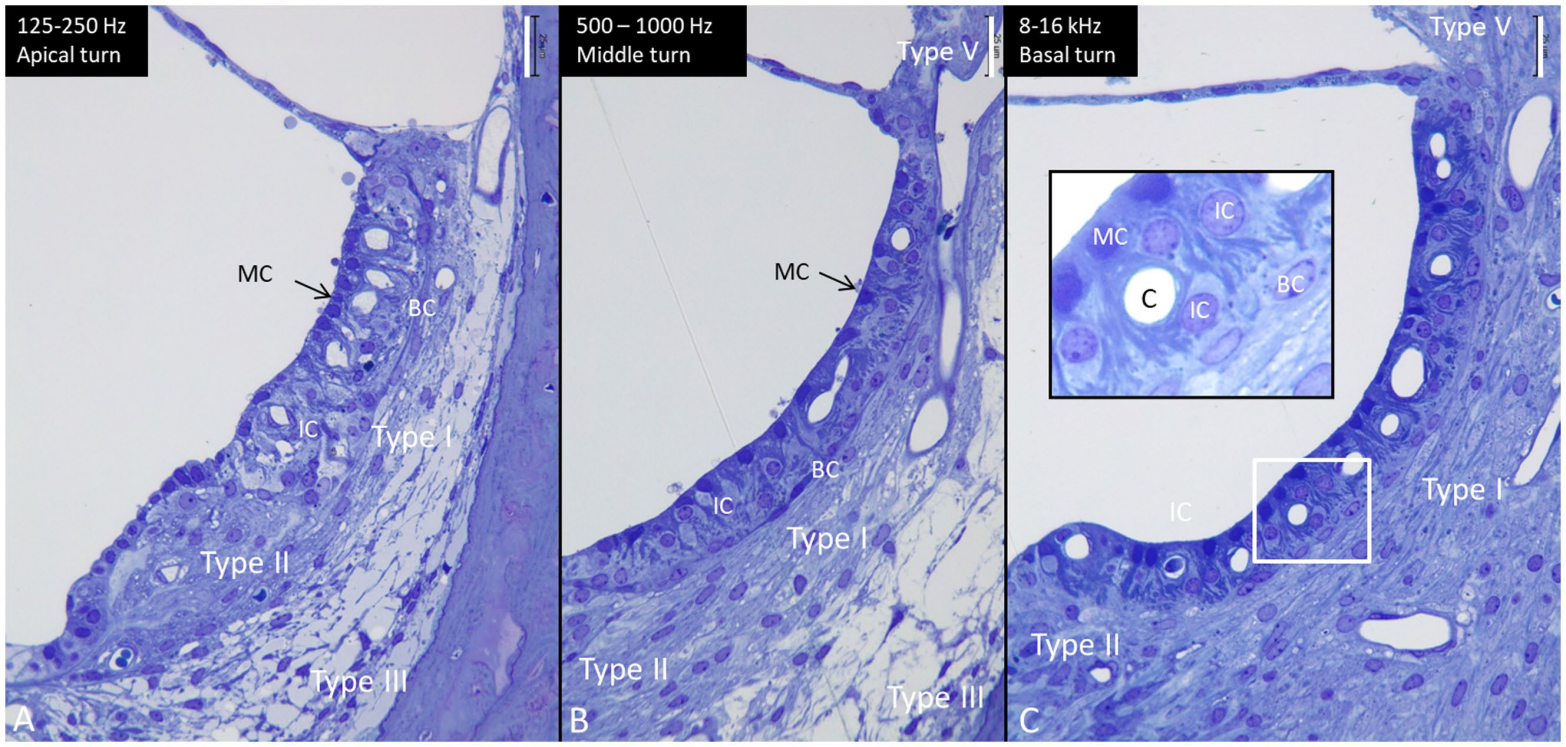
Semi-thin sections of the lateral wall at the three turns of the human cochlea (same magnification). The length of the stria vascularis in the apex **(A)**, mid-turn **(B)**, and base **(C)** is 132 microns, 204 microns, and 244 microns, respectively. Type I, II, III, and V fibrocytes are visualized. BC, basal cell. IC, intermediate cell. MC, marginal cell. Inset in C shows the framed area in higher magnification. MCs show many basal enfoldings. Staining with toluidine blue and osmium tetroxide. Scale bar is 25 μm.

**Figure 7:**
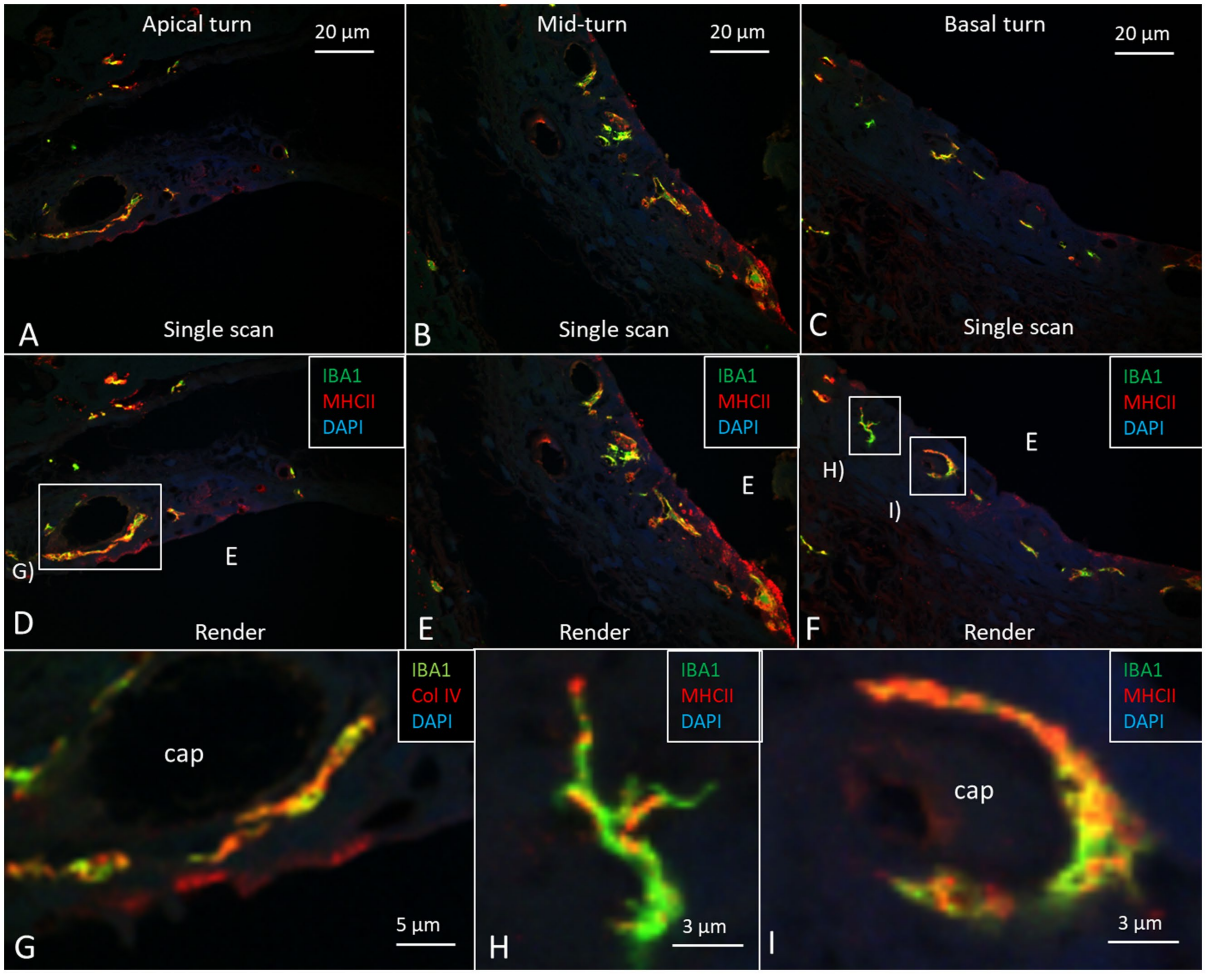
Confocal microscopy images show IBA1/MHC II immunohistochemistry in the three cochlear turns with single scans **(A–C)** and rendering **(D–F)**. Most perivascular IBA1 cells also express MHC II in the apical turn. Framed areas are magnified in **(G–I)**. **(G)** Perivascular IBA1 cell co-expressing MHC II. **(H)** A dendritic IBA1 cell expresses MHC II in the basal turn. I. Perivascular cells in the basal turn expressing both IBA1 and MHC II.

The cell architecture of the stria vascularis differed along the cochlear partition. Basally, the tri-cellular layer of marginal, intermediate, and basal cells was well-defined; apically, the arrangement of cells was less regular (Figure 6). Stria length varied from 132 μm apically to 244 μm basally at 8–16 kHz. There was no suprastrial tissue located most apically in the cochlea in this light-microscopy specimen. At the base, the marginal cells had a dark cytoplasm and displayed extensive basal enfolding reaching the blood capillaries and intermediate cells (Figure 6C, inset). The intermediate cells had a cubic shape with light cytoplasm and large round nuclei. These cells did not morphologically represent the slender IBA1 cells present in the stria vascularis. Figure 7 shows confocal microscopy of cells co-expressing IBA1 and MHC II in the three turns. Perivascular cells in the stria vascularis co-expressed IBA1 and MHC II. MHC II was mostly membrane-associated but also seemed intra-cytoplasmic (Figures 7G–I).

**Figure 8:**
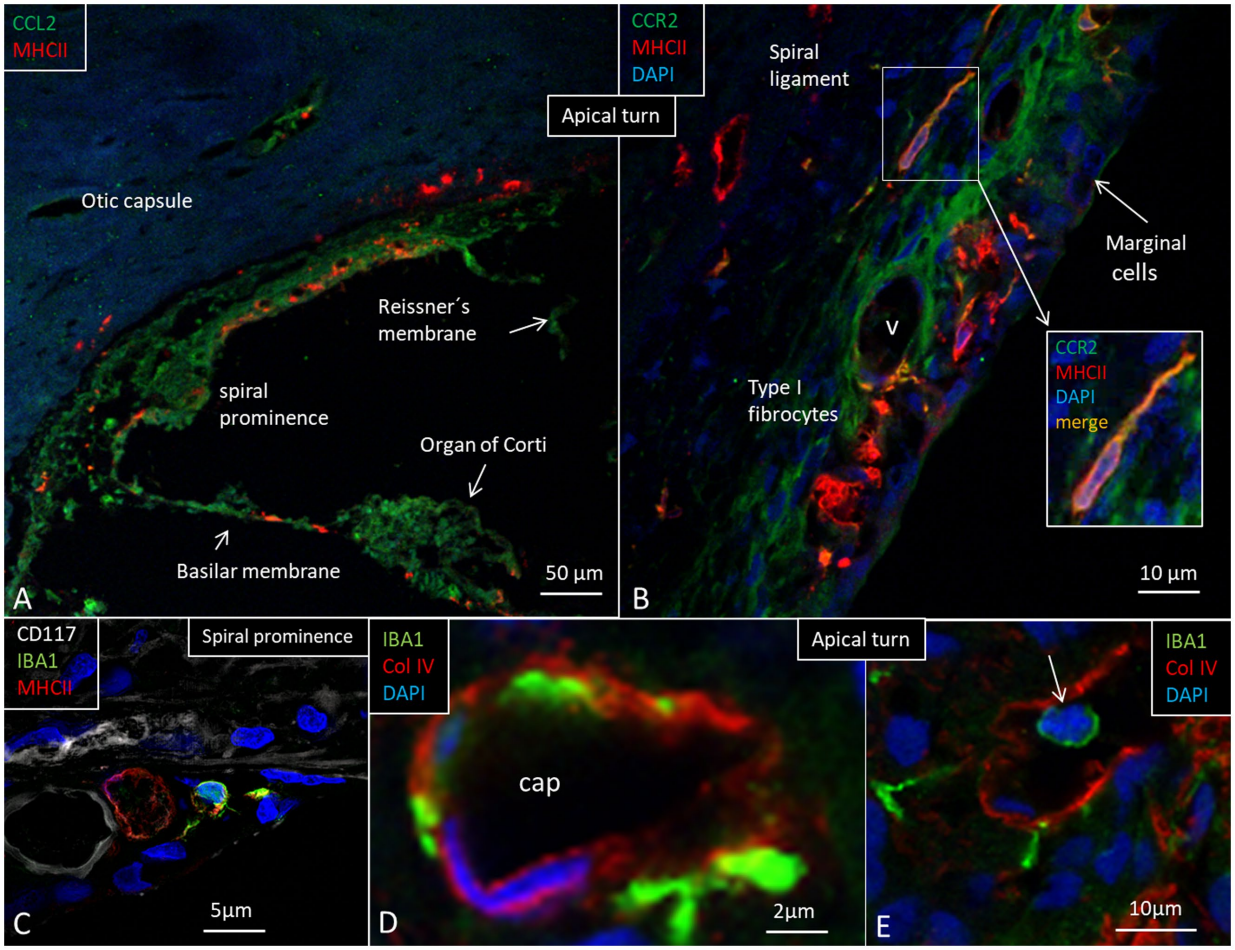
**(A)** Expression of chemokine C-C motif ligand 2 (CCL2), monocyte chemoattractant protein-1. **(B)** Expression of C-C chemokine receptor type 2 protein (CCR2) in the apical turn. Some cells bordering the apical stria region show faint immune staining with strong MHC II expression in cells at the intermediate portion. **(C)** IBA1 cells at the spiral prominence together with cells expression CD117 and MHC II. **(D)** Stria vessels demonstrating collagen IV expression with IBA1 cells located in the wall of the vessel as well as subendothelially. **(E)** A stria blood vessel contains an IBA1-positive cell (arrow).

At the base, the spiral ligament was larger, more cell-rich and well-vascularized (Figure 6) with occasional dendritic- and monocyte-like cells with folded electron-dense nuclei. In the spiral ligament, macrophages were found among the type II and V fibrocytes, but rarely amid type I fibrocytes. A few cells among the type III fibrocytes expressed IBA1. The total number of IBA1 expressing cells appeared to be highest in the mid-frequency region. A few isolated CD4 and CD8 cells were identified in the spiral ligament (not shown). There was diffuse expression of CCL2 in the lateral wall, while a few MHC II expressing cells seemed to express CCR2 (Figures 8A,B). CD117 was expressed in some cells in the spiral prominence (Figure 8C). IBA1 cells lay close to and penetrated the capillary basal lamina (Figure 8D). Some stria vessels contained IBA1 cells, assumingly representing migrating blood monocytes (Figure 8E).

### The spiral lamina, limbus and basilar membrane

**Figure 9:**
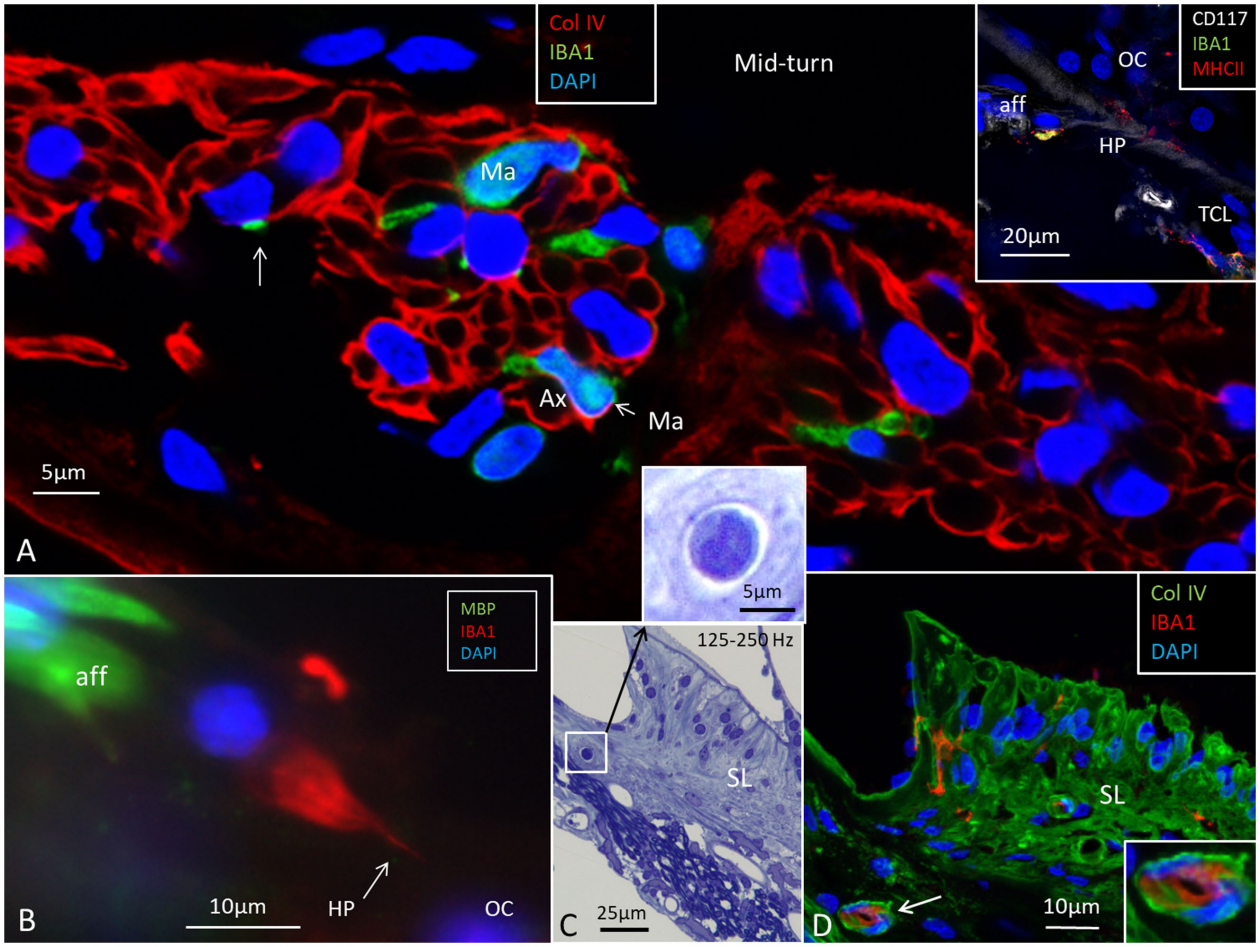
Expression of IBA1-positive cells in the spiral lamina and spiral limbus. **(A)** Several macrophages (Ma) are located among the population of neurons. Inset shows a CD117-positive cell in the tympanic covering layer (TCL) near the habenula perforata (HP). **(B)** A thin process from the IBA1 cell extends into the habenula perforata and organ of Corti (OC). **(C)** Semi-thin section of the limbus shows a capillary housing a monocyte-like cell. **(D)** Immunohistochemistry shows a similar located vessel containing IBA1 cells. The spiral limbus also contains several IBA1 cells.

At low-and mid-frequencies, the thin basilar membrane was covered underneath by a tympanic covering layer (TCL) consisting of free cells and a few blood vessels in an extra-cellular ground substance. Blood vessels placed near the habenula perforata were surrounded by lymphocyte- and monocyte-like cells. In the lower basal turn, the TCL was thin and contained few free cells. The basilar membrane was thicker there and consisted of a radial fibrous layer and a homogenous extra-cellular layer. Surprisingly, they formed a “zona arcuata-” and “zona pectinata-like” structure similar to the basilar membrane described in the mouse and guinea pig (38). Occasionally, Reissner’s membrane displayed single IBA1 cells. The TCL (except the most basal part) contained IBA1-positive cells. A few IBA1 cells could be detected in the organ of Corti. The spiral lamina contained many IBA1 cells (Figures 9A,B). They interacted with myelinated axons, and their cell processes frequently projected deep into the Schwann cell layer. Among the myelinated axons, several IBA1-positive cells co-expressed MHC II. At the habenula perforata, IBA1 cell processes seemed to extend into the organ of Corti as earlier demonstrated (4) (Figure 9B). CD117 cells occurred in the TCL beneath the basilar membrane (Figure 9A, inset) except basally near the round window. Light microscopy of a vessel containing a monocyte-like cell is shown at the spiral limbus in the 125–250 Hz region in Figure 9C. Immunocytochemistry of the same region shows a corresponding vessel containing an IBA1-positive cell (Figure 9D). Cells in the TCL and organ of Corti expressed fractalkine (not shown). There were no fractalkine gene transcripts expressed in the spiral lamina nerve fibers, but they were found in cells surrounding the unmyelinated nerve bundles beneath the habenula perforata as earlier demonstrated (4). There was a bony secondary spiral lamina (SSL) separating the spiral ligament from the scala tympani at the round window.

### SR-PCI and the round window niche

**Figure 10:**
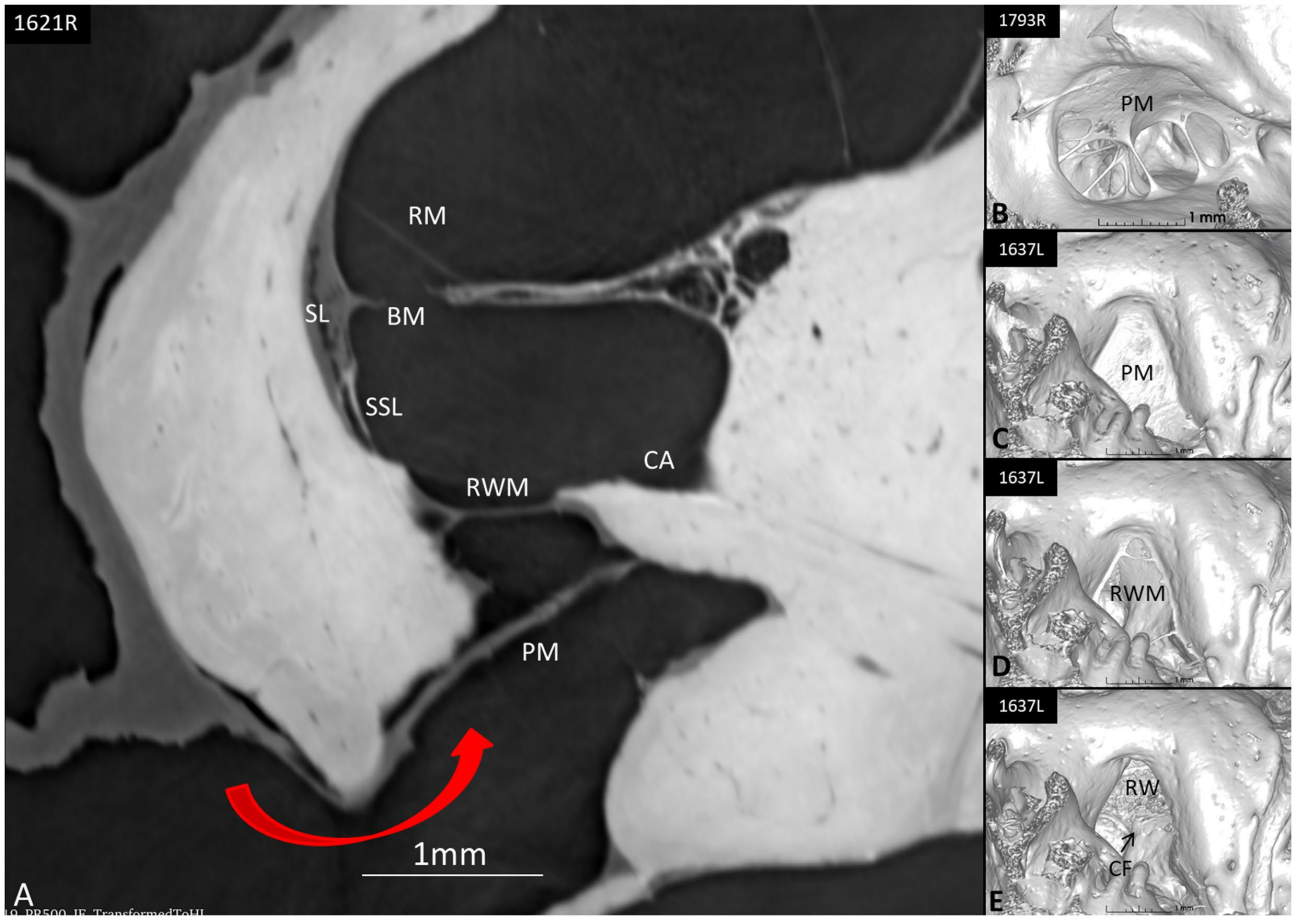
**(A)** Synchrotron section of the round window niche (red arrow at opening) showing possible protection mechanisms such as the shield of the pseudo-membrane (PM) that completely conceals the round window membrane (RWM). **(B)** 3D reconstruction of a round window niche with a web-like pseudo-membrane partly sealing the niche. **(C)** The PM incompletely conceals the niche. **(D,E)** show the RW niche with scalar opacity adjusted to reveal/hide RWM and PM, exposing the different compartments before reaching perilymph space. CF, crista fenestra; BM, basilar membrane; SSL, secondary spiral lamina; CA, cochlear aqueduct; SL, spiral ligament; BM, basilar membrane; RM, Reissner’s membrane.

SR-PCI sectioning and 3D reconstruction of the RWN were performed using 10 specimens. The results showed that the external window area contained a false membrane more or less blocking the entrance to the round window (Figure 10). It was mostly incomplete but also fully isolated the window from the middle ear. Occasionally, the extra membrane was concave, bulged inwards, and partly fused with the central region of the RWN, thus forming an extra inter-membranous space external to the round window.

### SR-PCI and the vestibular aqueduct

**Figure 11:**
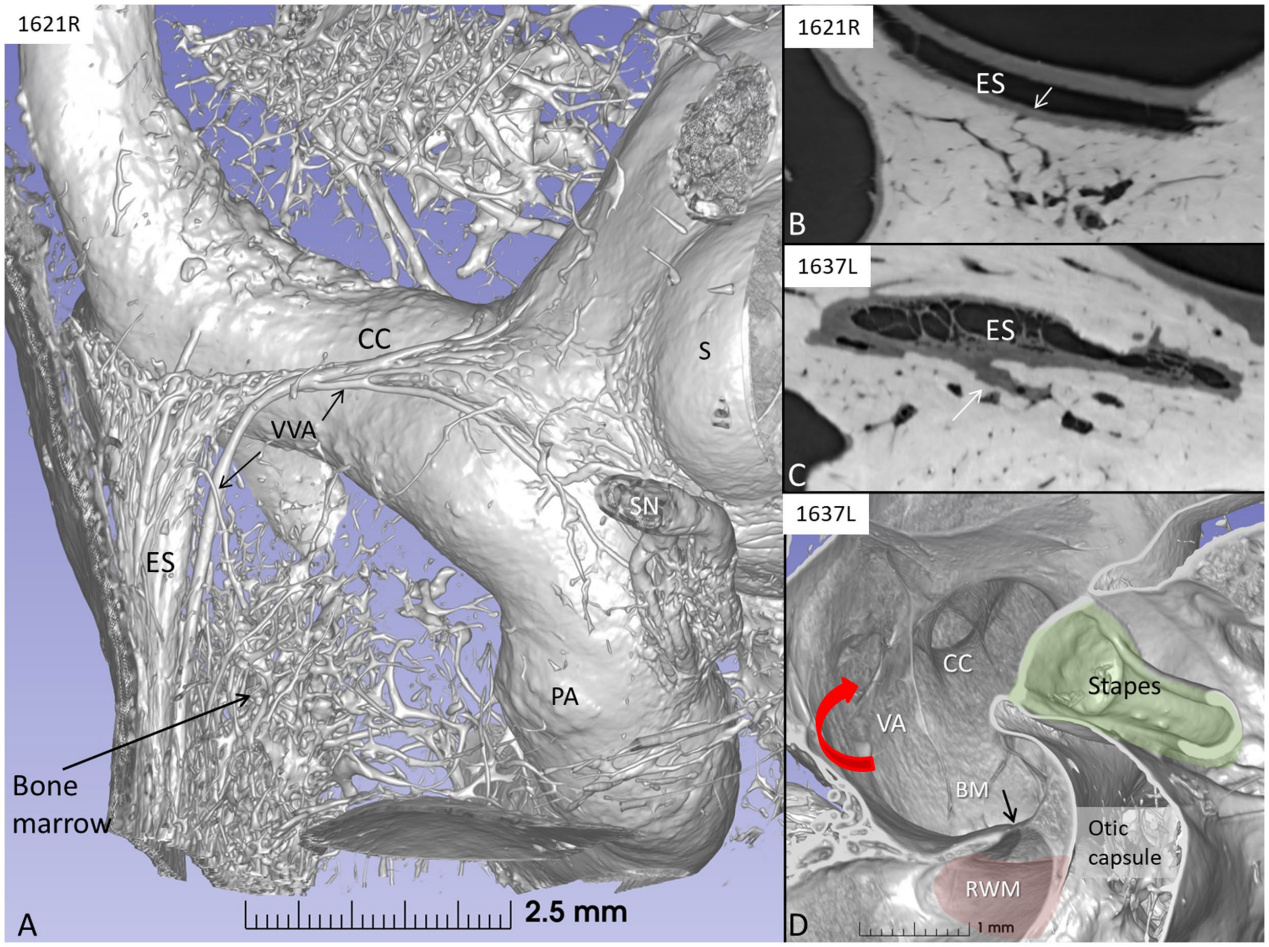
**(A)** SR-PCI and 3D reconstruction of a left human inner ear showing the extra-osseous endolymphatic sac (ES) and its relationship to the common crus (CC) and the periaqueductal bone marrow. **(B)** Section showing connecting vessels between the sac and bone marrow space. **(C)** Section of the intra-osseous ES. Bone marrow vessels merge with the perisaccular connective tissue. **(D)** Vertical section of the vestibule showing the internal aperture of the vestibular aqueduct (red arrow) at the medial wall of the vestibule. RWM, round window membrane; BM, basilar membrane (black arrow); PA, posterior ampulla; VVA, vein of the vestibular aqueduct; S, saccule; SN, singular nerve.

Synchrotron imaging demonstrated a close connection between the periaqueductal bone-marrow space and the endolymphatic sac with several communicating blood vessels and sinusoids. The number of vessels differed depending on the size of the intra-osseous portion of the sac. The bone channels interacted with the draining vein of the vestibular aqueduct (Figures 11A–D). The vessels may produce direct routes for immune cells into and out of the ES.

### Endolymphatic sac (ES)

**Figure 12:**
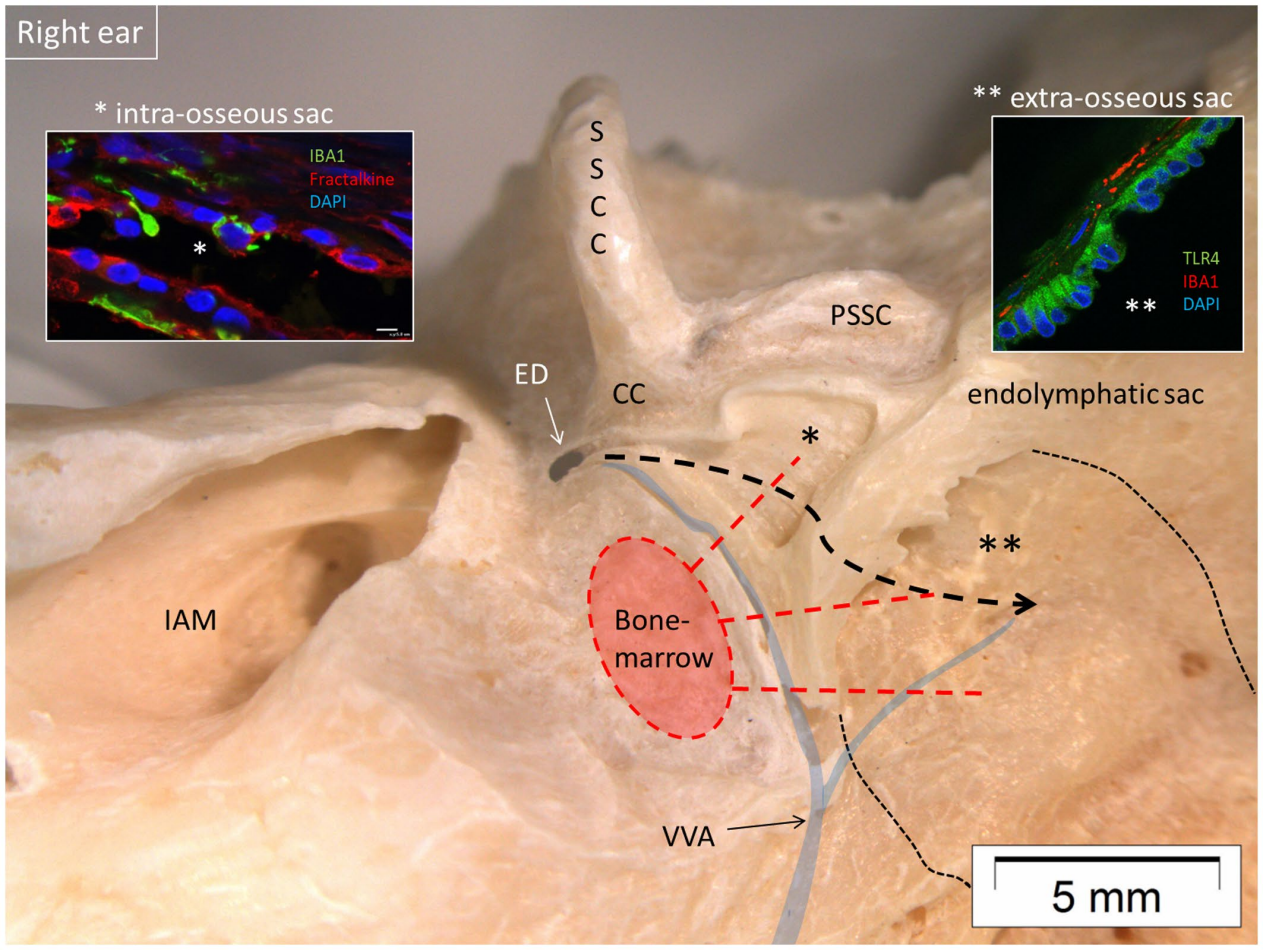
Micro-dissection of a right human vestibular aqueduct containing the endolymphatic duct and sac (posterior view). The sac contained IBA1 macrophages and lymphocytes suggesting an ongoing immune activity. Left inset shows the presence of IBA1 macrophages and expression of the chemokine fractalkine in the intra-osseous part of the sac (*). Right inset shows expression of TLR4 in the epithelial cells of the extra-osseous sac. Both parts are connected to the surrounding bone marrow by vascular bone channels. It is conjectured that antigens may reach the sac via the duct (bold interrupted line) highlighting its role in inner ear adaptive immunity. IAM, internal acoustic meatus; PSSC, posterior semicircular canal; SSCC, superior semicircular canal; VVA, vein of the vestibular aqueduct; CC, common crus; ED, endolymphatic duct; TLR4, toll-like receptor 4. Thin interrupted line: extra-osseous endolymphatic sac.

The sac contained a large population of IBA1 macrophages with migrant behavior, interacting with other immune cells, thus confirming the results obtained by Liu et al. (2) and Kämpfe et al. (35). IBA1 cells were found in the connective tissue, the epithelium, and the lumen of the sac (Figure 12). Some IBA1-positive cells in the epithelium seemed to be a part of the epithelium. Many of the IBA1 cells co-expressed MHC II. The epithelial cells expressed MHC II in the apical membrane but also contained endocytic vesicles expressing MHC II. A rich trans-epithelial migration in the intermediate portion of the sac was suggested by the irregular and fragmented collagen IV immunostaining of the basal lamina. Several migrating cells expressed CD68 and CD11b together with MHC II. Many epithelial cells of the tubules and sub-epithelial cells of the ES expressed toll-like receptor 4 (TLR4) in the cell membrane and cytoplasm (Figure 12, inset). CD4 and CD8 cells occasionally interacted physically with IBA1 positive cells. The sac epithelium and sub-epithelial cells expressed chemokine fractalkine and, occasionally, sub-epithelial fibrocytes. Fractalkine gene transcripts were not analyzed.

## Discussion

The present study demonstrates the wide variations in cellular anatomy in the human cochlear partition at different frequency locations. Our findings confirm that the human cochlea contains numerous IBA1-positive macrophages and that these are broadly distributed at all frequencies under steady-state conditions (1, 5). IBA1 cells occasionally co-expressed MHC II, indicating that these cells had been immunologically challenged. The human cochlea is likely surveilled by macrophages derived from activated resident cells or externally recruited monocytes differentiating into IBA1-positive macrophages. Surprisingly, a few CD4 and CD8 lymphocytes and even CD19 B-lymphocytes co-expressing MHC II were present in the spiral ganglion and spiral ligament but not inside the sensory epithelium of the organ of Corti as recently described (3). The MHC class II molecules present antigens to CD4 T-lymphocytes and are critical for the initiation of antigen-specific immune responses (39). CD19-positive B cells expressing MHC class II can act as antigen-presenting cells and amplify CD4 responses to T cell-dependent antigens (36, 39). The cells have also been associated with the development of autoimmune disease (37). The human cochlear nerve and spiral ganglion contained IBA1-positive macrophages of different phenotypes. They physically contacted ganglion cell bodies and showed occasional terminal dilations, reminiscent of synaptic contacts. Macrophages formed contact points at perineural spaces and possibly even at Ranvier’s nodes. Axons and dendrites interacted physically with macrophages at all frequencies. Their long processes or “antennae” (width of 0.1–0.2 μm) adhered to surrounding cells, suggesting migratory properties and a surveillance or scanning function along the nerve fibers. Type I ganglion cell bodies expressed fractalkine, and the RNAscope technique revealed CX3CL1 gene transcripts. Macrophages were earlier found to express CX3CR1, suggesting a CX3CL1-CX3CR1 or fractalkine signaling pathway in the human cochlea (4). This pathway may offer protection under steady-state conditions as earlier proposed experimentally (16, 40). In the brain, microglia and monocyte-derived macrophages are known to mount immune responses and are essential for neuronal regulation, synapse organization, and clearance of scavengers and toxic products (41–45). Ramified IBA1 cells, such as those observed in the human cochlea, may fight pathogens (46, 47) and respond to cell injury (48). In the lesioned brain, activation of non-astrocytic cells may transform into amoeboid-type microglia (49). Similarly, experimental damage to the inner ear elevates the number of macrophages in the auditory nerve, spiral ligament, and limbus (9, 16, 44, 50–52). Interestingly, there is evidence of increasing macrophage interactions with the myelinated axonal projections and type I spiral ganglion cell bodies in the human cochlea with age (19). This may suggest that abnormal macrophage/glia interactions may be linked to age-dependent auditory nerve degeneration. In the central nervous system, the importance of microglia and macrophages in the regulation and preservation of myelin and cognition has recently been highlighted (53). There is speculation that the effects of anti-inflammatory drugs can even reduce harmful microglial responses in connection to neuro-inflammation (43).

A remarkable characteristic of the human auditory nerve is the un-myelination of type I ganglion cell bodies, including their axonal initial segments. Instead of a compact myelin layer, which is essential for fast nerve conduction, thin satellite cells enwrap the cell bodies. This may reflect a different signal processing in humans and could facilitate a direct physical interaction between macrophages and axons. SR-SIM revealed gracile macrophage processes directly facing the neuronal cell membrane. Considering the potential of macrophages to support regeneration and neurotrophin stimulation (54), this may be significant in maintaining human auditory nerve integrity under various conditions. It may also partly explain the robustness of the human nerve following loss of hair cells and peripheral axons caused by noise, ototoxic drugs, or aging (55), and it could also clarify the long-term effects of cochlear implants (CIs) (56). Obviously, more work is needed to explain fully the role of fractalkine signaling in the human cochlear nerve. The immune cells in the human auditory nerve may participate both in the removal of damaged cells, repair and protection.

### Macrophages in the organ of Corti and the effects of noise

The present study confirms that macrophages may reach the sensory cell areas of the auditory and vestibular epithelium in assumed repair processes playing a role in organ homeostasis (4–6, 57). We found migratory macrophages near injured hair cells, possibly recruited to dispose of damaged cells (1). Wasted hair cells seem to retract beneath the reticular lamina and to be removed by activated IBA1-positive macrophages. This was supported by scanning electron microscopy that showed dendritic cells enclosing holes in the reticular lamina (4). Moreover, SR-SIM showed macrophages on the basilar membrane facing the organ of Corti co-expressing IBA1 and MHC II. If fractalkine signaling varies in its extent at different frequency locations in the human cochlea was difficult to prove. It could explain the increased vulnerability of the high-frequency sensory region.

A homing capacity of bone marrow-derived CD45 and CD68 macrophages to the spiral limbus was demonstrated after acoustic trauma (58). High-throughput RNA sequencing and real-time quantitative reverse transcription PCR (qRT-PCR) arrays showed strong expression of inflammatory genes in the organ of Corti associated with acoustic stress. This response was believed to originate from supporting cells that constitutively express immune genes and not from sensory cells (59). This may be linked to toll-like receptor signaling genes that are able to respond to acoustic trauma associated with sensory cell damage. The basilar membrane displayed distinct immune protein expression, monocyte infiltration, and transformation in the apical and basal sections of the cochlea. Only the basal monocytes and macrophages displayed increased expression of MHC II and class II trans-activator (CIITA), a MHC II production cofactor (60). They also showed that monocytes undergo a time-dependent transformation into macrophages after acoustic overstimulation together with CD4-positive T cells in antigen-presenting activity in the basilar membrane. It is reasonable to assume that a similar response in the TCL may be established in the apical and middle turns of the human cochlea after acoustic stress. At an early phase after noise exposure, IL-6 expression in SGNs, spiral ligament and stria vascularis were noted before macrophage activation (52). Anti-inflammatory cytokine therapy, including IL-6 blockade and antioxidant treatment, was suggested as a possible treatment for acute sensorineural hearing loss caused by noise (52, 61). Intriguingly, a lack of fractalkine signaling in CX3CR1 knockout animals resulted in an impairment of the repair of injured ribbon synapses after noise trauma (a model of “hidden hearing loss” or auditory synaptopathy), including auditory nerve degeneration (62). The authors’ results indicate that macrophages are involved in the recovery of ribbon synapses where lack of a receptor on the macrophages weakens the response. Further studies to explore the functional role of these neural macrophages in connection with human sensorineural deafness seems mandatory (40).

### Protection of the lateral wall of the human cochlea

The human stria vascularis forms a unique vascularized, tri-cellular epithelium, consisting of marginal, intermediate, and basal cells. Together with the adjacent fibrocyte layer, especially well developed in the basal cochlea, these cells form an exceptional endo-cochlear potential (EP) through a complex system of richly expressed ion channels, transporters, and isolator proteins (63, 64). The present investigation demonstrated the stria vascularis had a more organized cell architecture and larger volume in the middle and basal turns. Its size was almost double in the base compared with the apex. Spiral ligament volume difference might be even larger, possibly more than 10-fold, which could have functional relevance. This may influence K^+^ concentration and trans-epithelial endolymph transport (65–68). Despite the smaller size and reduced suprastrial tissue in the apex, the relative number of macrophages was large in the apex. A reliable quantitative analysis was difficult to achieve due to the limited number of available sections. Variances in lateral wall size and cell structure could challenge the idea that an equally high EP exists in the human cochlear turns. A gradient in EP, decreasing from cochlear base to apex, has been observed in several species (65, 67, 69, 70). Whether this relates to the physiological grounds of hair cell tuning and low-frequency coding is unknown (71, 72). Collectively, this could indicate that the EP generation is more prominent in the base that spreads apically, conceivably to fulfill high-frequency dependent outer hair cell electro-motility and place-coding. If so, an increased vulnerability of the base could also influence cochlear function at lower frequency regions.

Some macrophages showed thin cell processes that extended between marginal cells, possibly acting as immune sensors monitoring endolymph homeostasis (73). Nevertheless, the perivascular distribution of macrophages in all three turns suggests that these cells play an equally important role, presumably related to regulation of the blood–labyrinthine barrier and immune surveillance and protection in the entire stria vascularis. Earlier reports show the morphologic variety of macrophages ranging from thin, slender “antenna”-like processes in the auditory nerve, suggesting surveillance-mode as well as branched, amoeboid types often encountered in the lateral wall, and interacting with the strial vessels (1, 19). Our results show that both types of morphology exist normally in humans. Intriguingly, activated macrophages were observed in the basal turn of aged cochleae with an increased number of macrophages with reduced cellular processes in the middle and basal turns of the spiral ligament (19). Acoustic trauma was also found to increase cochlear inflammatory responses with recruitment of circulating leukocytes and upregulation of inflammatory mediators, chemokine, and cytokine in the spiral ligament. This also included immunohistochemical expression of the adhesion molecule ICAM-1 in the venules and endosteal cells of the scala tympani (44, 74). These responses seem to act at different time sequences to activate resident CX3CR1/macrophages and lateral wall fibrocytes with subsequent infiltration of immune cells (74). Invading immune cells after acoustic overstimulation included CD45, CD68, stromal-derived factor 1, F4/80, Iba-1, CD11b, and CX3CR1-positive macrophages (8, 44, 58). Yoshida et al. (75) speculated about a network of inflammatory cells, fibrocytes, and vascular endothelial cells interconnected by chemokines and mediators. Such a network could be therapeutically targeted to suppress inflammation and tissue damage (75) and seems to be supported by the present findings. The increased interest currently directed to spiral ligament fibrocytes is motivated by the potential targets for therapy in hearing loss and Meniere’s disease, including cell transplantation and genetic engineering (76). We found indications of monocyte chemoattractant protein-1 CCL2, a member of the CC family of chemokines, weakly expressed in the spiral ligament, basilar membrane, and organ of Corti. The chemokine receptor CCR2 was present in spiral ligament fibrocytes and was co-expressed with some MHC II cells. CCR2 was earlier found to be protective for inner ear hair cells after acoustic overstimulation independent of CCL2, although neither seemed to be necessary for monocyte migration (51). Similarly, spiral ligament fibrocytes may secrete CCL2 which has been found to be responsible for inner ear inflammation and monocyte attraction in experimental otitis media (77). Further analyses are needed to verify these preliminary findings in humans.

The spiral prominence showed rare cells expressing CD117 (KIT). Recently, mast cells were localized in the mouse and rat modiolus, spiral ligament, and stria vascularis using antibodies against c-Kit/CD117 (78). Their role in cochlear development, homeostasis, and pathology was discussed. Their function in the human lateral wall remains unknown and further analyses at different regions are necessary.

### Macrophages and human blood–labyrinth barrier

Here, we confirm that human stria vessels are partly surrounded by branched or amoeboid macrophages, which appear to be monocyte-derived (10). They may control the exchange between blood and interstitial space, as suggested by others (79–81). In addition to producing the EP, the stria represents an analogous “blood–labyrinth” barrier that serves to protect the inner ear under various conditions, such as inflammation and aging. The remarkable plasticity in macrophage responses has been documented (8, 74, 82–85). Macrophages did not seem to contain melanin granules, suggesting that they are unrelated to intermediate/melanocyte cells (1). The intermediate cells express potassium channels (Kir4.1), which are essential for the generation of the EP (63, 86–90). This contrasts with studies showing perivascular macrophage/melanocytes co-expressing both macrophage and intermediate cell markers F4/80 and Kir4.1 (81). The reason for these discrepancies may be species related. Using high resolution microscopy, the human intermediate cells were identified near vessels and showed a “light cell” appearance, with large round cell nuclei, different from the macrophage phenotypes. The potential role of microglia–vascular interactions to regulate blood–brain barrier integrity have been highlighted (91). Macrophages could similarly play an essential role in maintaining the blood–labyrinth barrier but also have a sentinel immune defensive role in controlling the trans-endothelial passage of various substances into the endolymph. A broken barrier caused by several conditions can lead to serious functional consequences, and therapies to reconstitute it are considered to prevent such consequences. *In vitro* studies have shown that fibrocytes can secrete chemokines and other mediators after stimulation of the pro-inflammatory cytokine TNF-α or IL-1β. This may extend the inflammation, induce fibrocyte damage, and impair EP production. Disturbed immuno-surveillance may influence the host defense system and result in increased microbial infections that could lead to functional disturbances and immune-pathologic conditions (92).

### Human round window shield

**Figure 13:**
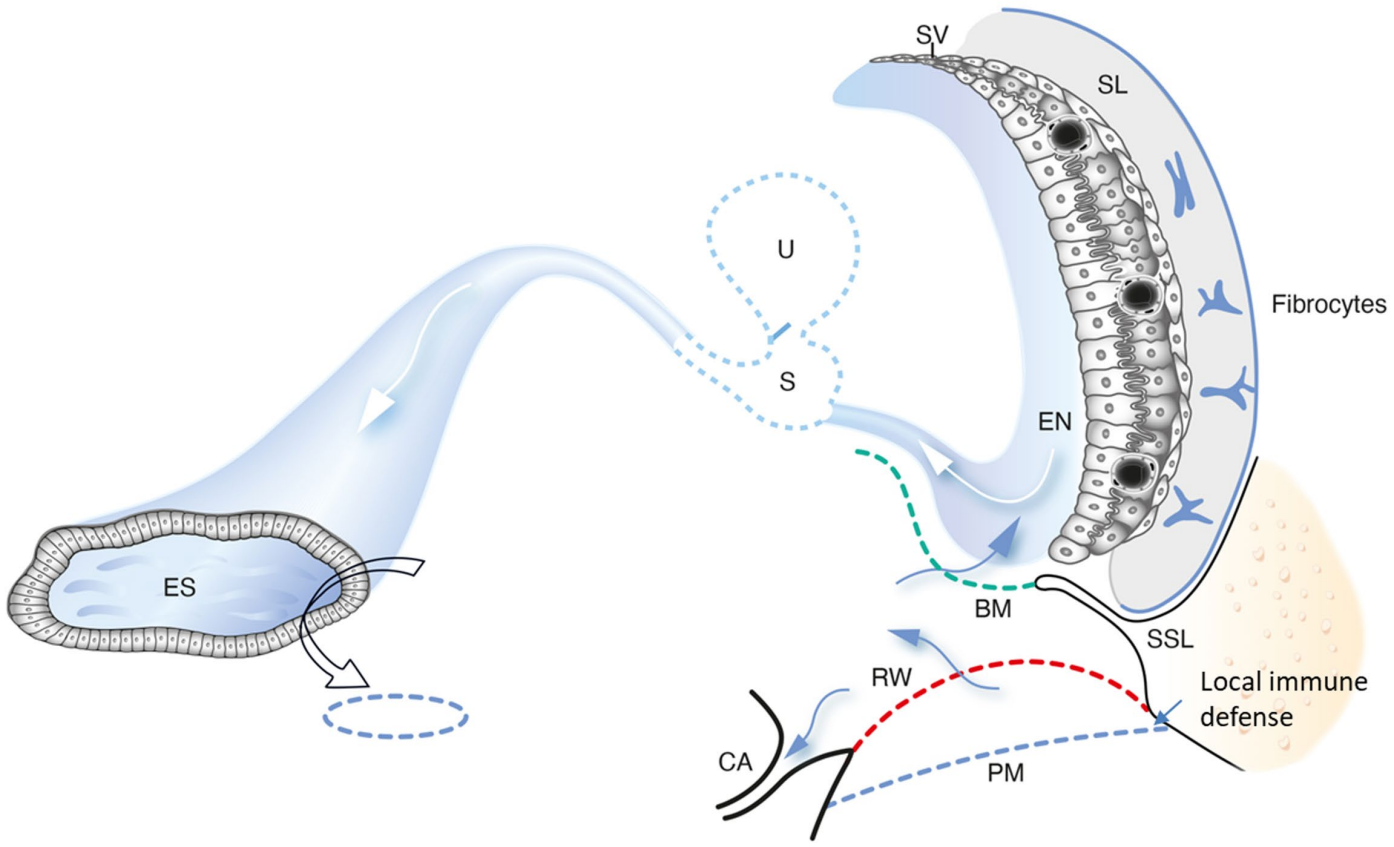
A schematic drawing showing conceivable trajectories for noxious substances to reach the human cochlea via the round window. A possible evasion along the duct system to the endolymphatic sac (ES) may avoid full-scale immune responses near the sensory structures and stria vascularis. The ES could play a key role in processing antigens for priming of immune cells to be recirculated to the inner ear. We conjecture that the periaqueductal bone marrow space plays a significant role to provide the ES with immune cells. A local mucosa immune defense at the BM also occurs. PM, pseudo-membrane. SSL, secondary spiral lamina. EN, endolymph; SL, spiral ligament. U, utricle; S, saccule; RM, Reissner’s membrane; BM, Basilar membrane; SV, Stria vascularis; CA, Cochlear aqueduct.

The inner ear immune cells may initiate immunological defensive cascades and protect the ear from invading pathogens, conceivably reaching across the RWM. This is considered a major route for bacterial toxins and inflammatory mediators in acute and chronic middle-ear infections, potentially affecting stria epithelium and the spiral ligament fibrocytes causing sensorineural hearing loss (93–97). The thickness of the human RWM is less than 0.1 mm, and its distance to the sensory cells is less than 1 mm (98, 99). Even though permeability of ions and macromolecules is normally restricted, which has been shown experimentally (96), the membrane may become permeable in otitis media, requiring additional protective measures (100, 101). This may involve mechanical barriers such as the round window niche (RWN) and “pseudo-membranes” sheltering the round window and organ of Corti. Studies have shown that the RWN anatomy is highly variable as it is either plugged with fibrous tissue or fat or covered by a false RWM. This may explain the varied permeability found in ears, which influences drug administration therapy to treat inner ear diseases via the middle ear (102, 103). The present study using SR-PCI and 3D reconstruction presented partly or completely shielded RWMs. Surprisingly, a cell-containing TCL was not evident in the most basal region of the cochlea to prevent noxious substances from reaching the sensory organ. Conceivably, this area could be protected by a thicker and more impermeable basilar membrane, a secondary spiral lamina, or drainage through the cochlear aqueduct constituting additional possible defense mechanisms (Figure 13). Remarkably, a local immune defense was discovered at RWM rim, in the cynomolgus monkey with gland-like structures, lymph channels, and sinusoidal veins containing leukocytes, plasma cells and monocytes (100). If a similar immune protective system occurs in humans is subject to investigation in our laboratory.

### Role of human endolymphatic sac in inner ear immunity

The results suggest that there is ongoing innate and adaptive immune activity in the ES in humans. The sac epithelium and peri-saccular tissue contain a large population of IBA1 co-expressing MHC II. In addition, migrating cells express CD68 and CD11b together with MHC II, TLR4 with CD4 and CD8 cells occasionally interacting with macrophages (35). The sac epithelium and sub-epithelial cells expressed fractalkine and, occasionally, sub-epithelial fibrocytes. The sac may receive antigens and waste material via the endolymphatic duct, which activates resident macrophages/monocytes including cells conceivably from the surrounding blood vessels (7, 104, 105). Synchrotron imaging showed vascular connections between the bone marrow and the sac that varied in number; temporal bones with a large sac displayed more interconnections. A close functional relationship between perisaccular bone marrow-derived macrophages and the ES was earlier described experimentally (106, 107). These cells are believed to migrate into the sac lumen followed by phagocytosis, and may return to the perisaccular tissue for antigen presentation to CD4-positive T-cells and immune processing. These responses may be amplified by MHC class II molecules, TLR4, and IFNγ stimulation. Møller et al. recently showed TLR4 and TLR7 expressed on the luminal side of the sac epithelium, suggesting the ability to identify and trap bacterial antigens and virus RNA within the endolymphatic space (108). Conceivably, immune cells may reach the sac attracted by chemokine fractalkine signaling. From there, they may recirculate to “prime” the inner ear tissue acting as perivascular “doormen” in the metabolically hyperactive stria vascularis and spiral ganglion tissue. This organization, away from the receptor areas, voids a full-scale immune response with the release of damaging pro-inflammatory mediators and antimicrobial activity near the vulnerable sensory cells. Whether sac size variations influence the immune capacity in different individuals is unknown. Notably, a smaller ES and vestibular aqueduct have earlier been shown in patients with Meniere’s disease (109, 110). Protection and possible trajectories for pathogens and noxious substances to reach the cochlea and ES under various circumstances are shown in Figure 13.

### Limitations of this study

Although there were no signs of tumor invasion, all patients had pathology in the vicinity of the inner ear that could have influenced the composition of the immune cells. Due to the small collection of the unique cochlear specimens, the number of control staining was limited. Furthermore, the endolymphatic sac was removed in patients with acoustic schwannoma, a condition that could influence inner ear fluid homeostasis and immune conditions. The sac removed at petro-clival meningioma surgery showed similar results, where there were no obvious inner ear changes.

## Data availability statement

The datasets presented in this article are not readily available because the synchrotron data cannot be publicly shared. Requests to access the datasets should be directed to the corresponding authors.

## Ethics statement

The studies involving humans were approved by the Uppsala University Hospital (no. 99308) Ethics Review Board (no. 99398, 22/9 1999, cont., 2003, no. C254/4; no. C45/7 2007, Dnr. 2013/190). The studies were conducted in accordance with the local legislation and institutional requirements. The participants provided informed consent to participate in this study. Cadaveric samples used for SR-PCI were obtained with permission from the body bequeathal program at Western University (London, ON, Canada) in accordance with the Anatomy Act of Ontario and Western’s Committee for Cadaveric Use in Research (Approval #06092020). Part of the research described in this paper was performed at the Canadian Light Source, a national research facility of the University of Saskatchewan. The studies were conducted in accordance with the local legislation and institutional requirements. The participants provided written informed consent to participate in this study.

## Author contributions

WL: Investigation, Methodology, Software, Visualization, Writing – review & editing. HOL: Data curation, Investigation, Methodology, Software, Visualization, Writing – review & editing. CK: Investigation, Methodology, Visualization, Writing – review & editing, Conceptualization. ND-L: Investigation, Methodology, Writing – review & editing. SA: Methodology, Investigation, Writing – review & editing, Conceptualization, Data curation, Supervision, Validation, Visualization. HML: Conceptualization, Data curation, Methodology, Supervision, Validation, Writing – review & editing, Funding acquisition, Resources, Software. HR-A: Funding acquisition, Supervision, Project administration, Writing – original draft.

## Glossary

c-Kit/CD117: Tyrosine-protein kinase KIT, cluster of differentiation 117 or mast/stem cell growth factor receptor
CD4: Cluster of differentiation 4: Glycoprotein co-receptor for the T-cell receptor
CD8: Cluster of differentiation 8: Glycoprotein co-receptor for the T-cell receptor
CX3CL1: C-X3-C Motif ligand 1
CX3CR1: C-X3-C Motif Chemokine Receptor 1 (“fractalkine receptor”)
DAPI: 4′, 6-Diamidino-2-phenylindole dihydrochloride
EDTA: Ethylene-diamine-tetra-acetic acid
EP: Endo-cochlear potential
ES: Endolymphatic sac
HP: Habenula perforata
IBA-1: Ionized calcium-binding adapter molecule 1,
IGSB: Intra-ganglionic spiral bundle containing cochlear efferents
MHC II: Major histocompatibility complex type II
MBP: Myelin basic protein
P2RY12: Purinergic receptor P2Y12 (microglia marker)
RWM: Round window Membrane
RWN: Round window Niche
SGNs: Spiral ganglion neurons
SL: Spiral limbus
SP: Spiral prominence
SR-SIM: Super-resolution structured illumination fluorescence microscopy
TCL: Tympanic covering layer
TLR4: Toll-like receptor 4
TMEM119: Transmembrane protein 119 (“microglia marker”)
Type I ganglion cells: Large afferent neurons innervating the inner hair cells
Type II ganglion cells: Small afferent neurons innervating the outer hair cells
